# Hot, Tired and Hungry: The Snacking Behaviour and Food Cravings of Firefighters during Multi-Day Simulated Wildfire Suppression

**DOI:** 10.3390/nu12041160

**Published:** 2020-04-21

**Authors:** Charlotte C. Gupta, Sally A. Ferguson, Brad Aisbett, Michelle Dominiak, Stephanie E. Chappel, Madeline Sprajcer, Hugh H. K. Fullagar, Saman Khalesi, Joshua H. Guy, Grace E. Vincent

**Affiliations:** 1 School of Health, Medical and Applied Sciences, Central Queensland University, Adelaide 5034, Australia; 2 Institute of Physical Activity and Nutrition (IPAN), School of Exercise and Nutrition Sciences, Deakin University, Geelong 3216, Australia; 3 Faculty of Health, University of Technology, Sydney 2007, Australia

**Keywords:** snacking, fatigue, bushfire, sleep

## Abstract

Firefighters are exposed to numerous stressors during wildfire suppression, including working in hot temperatures and sleep restricted conditions. Research has shown that when sleep restricted, individuals choose foods higher in carbohydrates, fat, and sugar, and have increased cravings for calorie dense foods. However, there is currently no research on the combined effect of heat and sleep restriction on snacking behaviour. Conducting secondary analyses from a larger study, the current study aimed to investigate the impact of heat and sleep restriction on snacking behaviour and food cravings. Sixty-six firefighters completed three days of simulated physically demanding firefighting work and were randomly allocated to either the control (*n* = 18, CON; 19 °C, 8 h sleep opportunity), sleep restricted (*n* = 16, SR; 19 °C, 4-h sleep opportunity), hot (*n* = 18, HOT; 33 °C, 8 h sleep opportunity), or hot and sleep restricted (*n* = 14 HOT + SR; 33 °C, 4-h sleep opportunity) condition. During rest periods firefighters were able to self-select sweet, savoury, or healthy snacks from a ration pack and were asked to rate their hunger, fullness, and cravings every two hours (eating block). Mixed model analyses revealed no difference in total energy intake between conditions, however there was a significant interaction between eating block and condition, with those in the CON, HOT, and HOT + SR condition consuming significantly more energy between 1230 and 1430 compared to the SR condition (*p* = 0.002). Sleep restriction and heat did not impact feelings of hunger and fullness across the day, and did not lead to greater cravings for snacks, with no differences between conditions. These findings suggest that under various simulated firefighting conditions, it is not the amount of food that differs but the timing of food intake, with those that are required to work in hot conditions while sleep restricted more likely to consume food between 1230 and 1430. This has potential implications for the time of day in which a greater amount of food should be available for firefighters.

## 1. Introduction

Wildland fires pose a significant threat to people and property, and frequently occur in Australia, North America, and Southern Europe [1,2]. Australia faces some of the most severe wildland fires, and consequently have some of the world’s largest fire and rescue agencies, consisting of many volunteer rural firefighters [3,4]. During wildfire suppression, firefighters can work extended hours over multiple days, and during this time experience a myriad of physical and emotional stressors, including extreme temperatures reaching 45 °C [5], rough terrain, sleep restriction, limited food availability, and smoke [4,6,7]. Of these stressors, heat and sleep restriction are particularly prevalent. 

During wildfire suppression, temperatures can range from mild to extreme 18–45 °C [5]. Performing physical work in hot conditions (30 °C) is linked to increased levels of acute stress, with studies showing increases in cortisol levels [8,9] and acute inflammatory responses [10,11]. The majority of research shows that the physical performance of firefighters is largely unaffected in hot conditions [12]. However, cognitive performance is negatively affected under hot conditions [13,14,15]. This is concerning given firefighters are often tasked with making safety critical decisions [4]. For example, firefighters must assess, react, and respond to hazardous situations, and impaired decision making can impact not only their own safety, but that of their crew members and civilians. 

During wildfire operations, firefighters are also required to work extended shifts, over multiple days [16], and during wildfire suppression this can result in restricted sleep [17]. Due to a number of factors such as shift start time, shift length, and sleeping in hot, smoky, and noisy conditions, some research has shown that firefighters can sleep on average for as little as 3–4 h per day [6,18]. While this level of sleep restriction can impair cognitive performance [13,14], it is also associated with greater risk of long-term health effects [19,20], with cardiometabolic disease prevalent among firefighters [21,22]. Further, the additive effect of sleep restriction in hot conditions has a synergistic effect on cognitive performance when compared with performance in hot or sleep restricted conditions alone [13,14], which has the potential to influence long-term health.

While the aforementioned body of research highlights some of the impacts of hot and sleep restricted conditions, what is unknown, is the influence of heat and sleep restriction on the food intake of firefighters during wildfire suppression. This is important to understand given that previous research in laboratory settings have found that food intake can influence on-shift cognitive performance [23] and can influence sleep quality and quantity [24]. For example, recent findings suggest that a large meal can negatively impact reaction time and sustained attention, two critical cognitive processes during wildfire suppression, compared to a smaller snack [25]. In addition, from a long-term health perspective, increased food intake on-shift, particularly at night, is linked to obesity due to consuming food at times when the body is not primed for digestion [23,26]. This can lead to chronic health conditions such as Type 2 diabetes [27] and cardiovascular disease [28]. 

Restricted sleep can impact food intake due to altered rhythms of hunger and appetite hormones [29]. Decreased levels of leptin and increased levels of ghrelin have been found following restricted sleep, leading to increased hunger and cravings for food during the day [30,31]. Further, the type of food consumed on-shift can also be influenced by sleep restriction. For example, shiftworkers have reported consuming fewer main meals and increased snacks when sleep restricted [23,25,32]. In addition, research examining food choices when sleep restricted have noted an increase in the consumption of carbohydrates [31,32,33], fat [34], and sugar [35]. Taken together, these findings suggest that, in sleep restricted conditions, workers may self-select snacks high in carbohydrates, fat and sugar. 

Compared to cold temperature, food intake may be less following physically demanding tasks in hot temperatures [36]. For example, studies have found suppressed appetite in hot temperatures compared to cooler temperatures [37,38,39]. In one study, there was no difference between energy intake post-exercise between hot and neutral temperatures [40]. This suggests that perhaps exercise impacts the relationship between heat and appetite. However, it is yet to be determined what the effect on appetite would be in hot temperatures after longer periods of physical activity, such as the activity that firefighters perform on-shift [16,41]. In addition to appetite, energy expenditure is increased in conditions of heat [42,43] and sleep restriction [44,45], and this is thought to impact energy intake [46]. Therefore, it is possible that the combined effect of heat and sleep restriction on energy expenditure may influence food choice for wildland firefighters.

The first step in understanding the impact of food on firefighters’ health and safety is to understand the food intake patterns of these firefighters, particularly when working in unique shift conditions. In a study of metropolitan-based Australian firefighters, food intake was largely determined by shift schedules, attitudes of co-workers, time, accessibility, and health [47]. However, these firefighters were not deployed in wildfire conditions, and sleep restriction and heat were not considered as potential influences on hunger and food choice. The individual and combined effect of sleep restriction and heat on food intake, hunger, and the type of food that firefighters are craving is yet to be determined. Knowledge of such interactions may help firefighting agencies to be better prepared to supply firefighters with ration packs suitable to different wildfire suppression conditions. Therefore, the aim of this study was to conduct secondary analyses investigating the impact of sleep restricted to 4-h, and hot conditions, on food and macronutrient intake during snack opportunities, and the subjective ratings of hunger, fullness, and cravings of firefighters during multi-day simulated wildfire suppression. 

## 2. Materials and Methods 

### 2.1. Participants

Volunteer and salaried rural firefighters (*n* = 66; 56 males) were recruited from state fire agencies across the southern states/territories of Australia (Victoria, South Australia, New South Wales, Australian Capital Territory, and Tasmania). Firefighters from northern states/territories were excluded due to their exposure to hotter average temperatures. Firefighters were also excluded if they were under 18 years of age, had a diagnosed sleep disorder, condition limiting their usual work (e.g., injury, illness, or pregnancy), or any contraindications to exercise. 

To limit the variation between the condition groups, firefighters were matched by age, sex, and body mass index (BMI) and then randomly allocated to one of four conditions; control (CON), sleep restricted (SR), hot (HOT), or hot and sleep restricted (HOT + SR). Participant demographics are presented in Table 1. There were no statistically significant differences between age or BMI between conditions [48], and the sample is broadly representative of wildland firefighter populations [18]. Data collection occurred from March to August 2012–2013 (autumn and winter months in Australia that are characterised by temperate to cool weather). All firefighters completed a medical questionnaire to ensure they were physically able to participate and subsequently provided written informed consent. Ethical approval was obtained from the Central Queensland University (H12/01-016) Ethics Committee and Deakin University (2010-170) Human Research Ethics Committee.

Data are presented as mean ± SD. CON = Control; SR = Sleep restricted; HOT = Hot; HOT + SR = Hot and sleep restricted.

### 2.2. Study Design

The current study was part of a large multidisciplinary project that investigated the impact of temperature, sleep, hydration, and stress on firefighters’ physical and cognitive performance during simulated wildfire scenarios. Details on methodology (including the pre-experimental protocol) and previously published research findings can be found here [8,12,48]. The methodology and measures specific to the current study are presented below.

**Figure 1:**
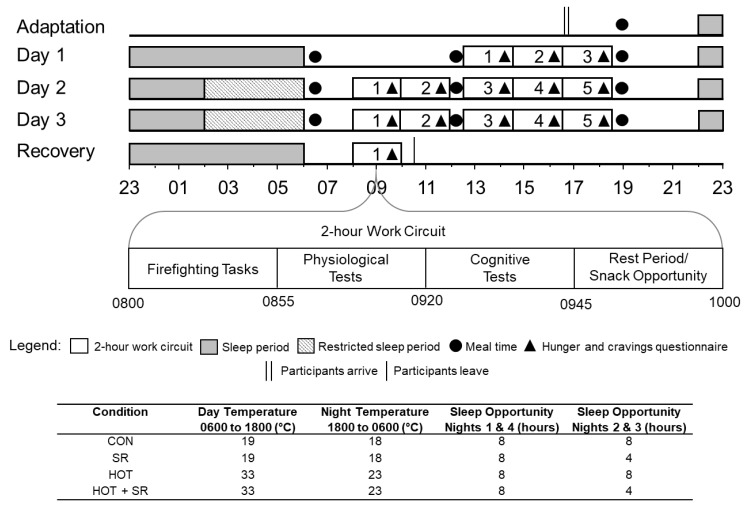
Experimental Protocol. CON = Control; SR = Sleep restricted; HOT = Hot; HOT + SR = Hot and sleep restricted.

Simulated wildfire scenarios took place in South Australia, Victoria, and Canberra. The study occurred across five consecutive days including an adaptation day, three experimental days, and a recovery day. Each trial was conducted in groups of 3–5 firefighters. To simulate a wildfire scenario, firefighters were required to ‘live in’ the laboratory for the duration of the study (except for showering and using the bathroom). Experimental conditions (e.g., ambient day and night temperatures and sleep opportunities) for each condition are shown in Figure 1. 

### 2.3. Experimental Procedures

Upon arrival to the laboratory, firefighters’ heights, measured with a stadiometer (Fitness Assist, Wrexham, UK), and semi-nude body mass, measured with calibrated electronic scale (A and D, Japan), were collected. During all trials, firefighters were required to wear their personal protective clothing to reflect real-life situations (i.e., two-piece jacket and trouser set, suspenders, boots, gloves, and helmet) which amounted to roughly 5 kg in weight, and were provided with a ration pack (3085 kJ) in addition to their daily meals.

The experimental protocol is provided in Figure 1. The first day of the experiment was the adaptation day, to familiarise the firefighters to study procedures. During the experiment days, firefighters followed a strict schedule for their meals, 2-h work circuits, and sleep opportunities. During the 2-h work circuits six physical firefighting tasks were performed. These tasks are used within real-world wildfire suppression (charged hose advance, blackout hose work, hose rolling, lateral repositioning rake, and static hold [48]), and are considered to be the most physically demanding and operationally important of the tasks performed during wildfire suppression work [49]. Previously published data indicate that there were no differences in physical tasks performed between conditions [12,17,18,48,49]. The details of these components of the 2-h work circuit, including descriptions of all tasks and work to rest ratios are reported elsewhere [13,17,18,49]. For Day 1 of the experiment, firefighters completed three 2-h work circuits (6h total). For Day 2 and 3, five 2-h work circuits occurred (10 h total). The final day was for recovery and included one more night of sleep and one 2-h work circuit, of which data is not reported.

In the CON and SR conditions, ambient temperature during the day (0600-1800) was 19 °C (40% relative humidity; RH) and during the night, temperature was 18 °C (40% RH). For the HOT and HOT + SR conditions ambient temperature during the day was 33 °C (40% RH), and during the night temperature was 23 °C (40% RH). Ambient air temperature was maintained throughout the simulated work shifts through the use of split cycle air-conditioners (Daikin Industries Ltd., Osaka, Japan) and portable ceramic disk heaters (Micro Furnace, Sunbeam, Adelaide, Australia). Both were measured and monitored continuously using a wireless temperature and humidity data logger, data receiver, and associated software (HOBO® Pro Software, One Temp Pty Ltd., Marleston, Australia). 

Smoking and caffeine consumption were restricted to the rest periods, while firefighters’ fluid consumption and snacking was *ad libitum* during rest periods. Post-dinner, firefighters engaged in free-time activity (e.g., read a book, watched a movie) until the sleep opportunity commenced. At all times, participants were monitored by researchers to ensure they did not fall asleep or perform physical work at nonspecified times. Participants slept on camp beds to replicate the sleeping conditions during a wildfire suppression deployment [48]. All sleep periods were recorded using standard polysomnography and for each period, total sleep time (min) was calculated. Sleep data is presented elsewhere [50,51]. Participants also wore activity monitors (*Actical* MiniMitter/Respironics, Bend, OR, USA) to measure sleep for the two days leading into the study. For further information on the methodology see [4,8]. 

### 2.4. Measures

#### 2.4.1. Snack Amount and Type

On each experimental day firefighter’s food and drink intake was recorded at each meal and during rest periods. Similar to a fireground setting, during rest periods participants ate *ad libitum* from a ration pack. This ration pack was 3085 kJ in total and contained a range of snacks identified by subject matter experts as similar to those available during a wildfire situation. The specific snacks provided are outlined in Table 2. Firefighters received a new ration pack for each day, and any leftover snacks were taken away (i.e., snacks did not accumulate). The snacking data was entered into FoodWorks 7 nutrition software (2012 Xyris Software Pty Ltd., High Gate Hill, QLD, Australia). Once the data was compiled in FoodWorks, the type and number of snacks ingested were extracted to identify the total energy intake from snacking, and then specifically for sweet, savoury, and healthy snacks. Macronutrient intake was also extracted from FoodWorks, including protein (g), total fat (g), and carbohydrate (g).

#### 2.4.2. Hunger

Firefighters hunger ratings were obtained using a hunger and cravings questionnaire that was developed for use in a similar study [35]. Firefighters completed this questionnaire as part of each cognitive performance testing battery (13 times total across the three experimental days), prior to eating any snacks. Firefighters were asked to identify their hunger and satisfaction levels on a 100mm point Visual Analogue Scale (VAS). Scores were integers between 0 and 100 (i.e., no decimal place values). Firefighters were asked four questions. (1) How hungry they felt on a scale of “I am not hungry at all” to “I have never been more hungry”, (2) how satisfied they felt on a scale of “I am completely empty” to “I cannot eat another bite”, (3) how full they felt on a scale of “not at all full” to “totally full”, and 4) how much they thought they could eat, “nothing at all” to “a lot”.

#### 2.4.3. Craving, Craving Type, and Craving Intensity 

The questionnaire also assessed firefighters’ cravings during each 2-h work circuit (13 times total across the three experimental days). Firefighters were asked an initial question to determine whether they were currently experiencing any desires/urges to eat specific foods. If yes was answered, firefighters were then asked to identify the type of food they were craving from a list; sweet carbohydrate/fat, savoury carbohydrate/fat, protein, or carbohydrate. Finally, firefighters were asked one question to rate the general intensity of their craving on a 100mm VAS scale, 0 (“very weak”) to 100 (“very strong”). The VAS score was an integer between 0 and 100 (i.e., no decimal place values), with a higher score representing a stronger craving intensity. 

### 2.5. Statistical Analysis

Data analyses were performed using IBM SPSS Statistics 25 (Armonk, New York). Prior to performing analyses, the data were screened for outliers, and no data were excluded based on this. There was missing data (*n* = 17) for the total intake data resulting in a final sample of *n* = 49 (CON *n* = 14; SR *n* = 16; HOT *n* = 18; HOT + SR *n* = 14). There was no missing data for the hunger and cravings data resulting in a final sample of *n =* 66 (CON *n* = 14; SR *n* = 16; HOT *n* = 11; HOT + SR *n* = 8). Linear mixed-effects analysis of variance (ANOVA) were used to determine if there were differences in total intake per day for total energy (kJ), carbohydrate (g), total fat (g), and protein (g), with fixed effects of condition (CON, SR, HOT, and HOT + SR), day (2 or 3), and condition × day, and a random effect of participant ID. Mixed effects ANOVAs were also used to determine if there were differences in total intake per food opportunity for total energy (kJ), carbohydrate (g), total fat (g), and protein (g), with fixed effects of condition (CON, SR, HOT, and HOT + SR), day (2 or 3), food opportunity block (1–5), condition × day, condition × block, and day × block, and a random effect of participant ID. Differences in hunger, fullness, and craving intensity were also analysed using mixed effect ANOVAs with fixed effects of condition (CON, SR, HOT, and HOT + SR), day (2 or 3), food opportunity block (1–5), condition × day, condition × block, and day × block, and a random effect of participant ID. Participant BMI was included as a covariate in all models. Due to the relative importance of the two-way interactions, the three-way interaction effects were dropped from the models. 

As cravings and cravings type were binary variables, a generalised estimating equation (GEE) was used with a binary logistic regression, with predictors of day (2 or 3), block (1-5), condition (CON, SR, HOT, and HOT + SR), condition × day, and condition × block reported (via Wald’s χ^2^). This analysis accounts for binary data with repeated measurements for each participant and adjusts for the effects of clustered sampling. 

Statistical significance was defined as *p* < 0.05 and post hoc pairwise comparisons were performed using a Bonferroni correction. The data analysed in the current study were secondary to the primary aims of the study, with primary outcomes published elsewhere [8,12,13,14,15,48,49,50,51,52,53,54,55,56,57].

## 3. Results

### 3.1. Total Intake Per Day from Snack Opportunities 

As seen in Table 3 there were no significant condition × day interactions for total intake per day on total energy, (kJ), carbohydrate (g), total fat (g), or protein (g). 

There were also no significant main effects of condition or day for total intake per day on total energy (kJ), carbohydrate (g), total fat (g), or protein (g; Table 4).

### 3.2. Total Intake Per Food Opportunity

**Figure 2:**
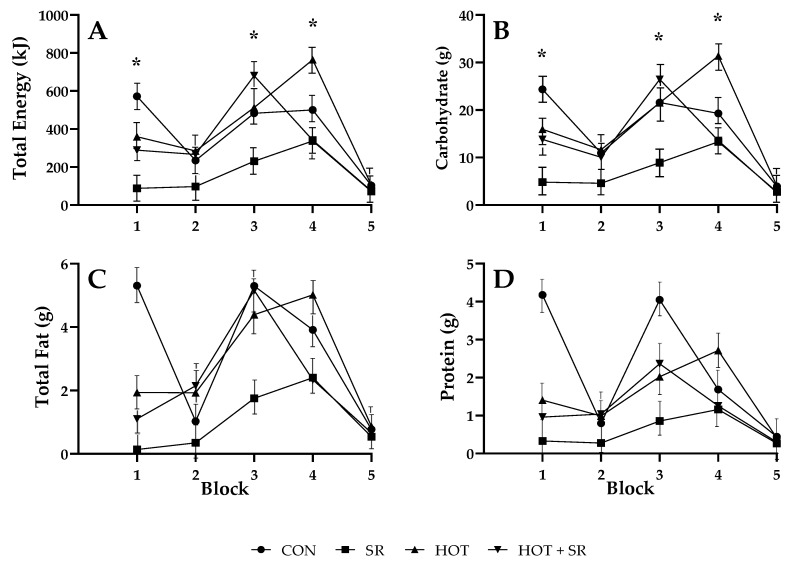
Total food intake per food opportunity across food opportunity blocks (1–5). A: total energy (kJ); B: carbohydrate (g); C: total fat (g); D: protein (g). CON = control condition. SR = sleep restricted condition. HOT = hot condition. HOT + SR = hot + sleep restricted condition. Block 1: 0800–1000; block 2: 1000–1200; block 3: 1230–1430; block 4: 1430–1630: block 5: 1630–1830. Lunch was provided at 1200 (after block 2) and dinner was provided at 1900 (after block 5). Error bars indicate standard error. Asterisk indicates a significant difference between conditions (*p* < 0.05).

**Figure 3:**
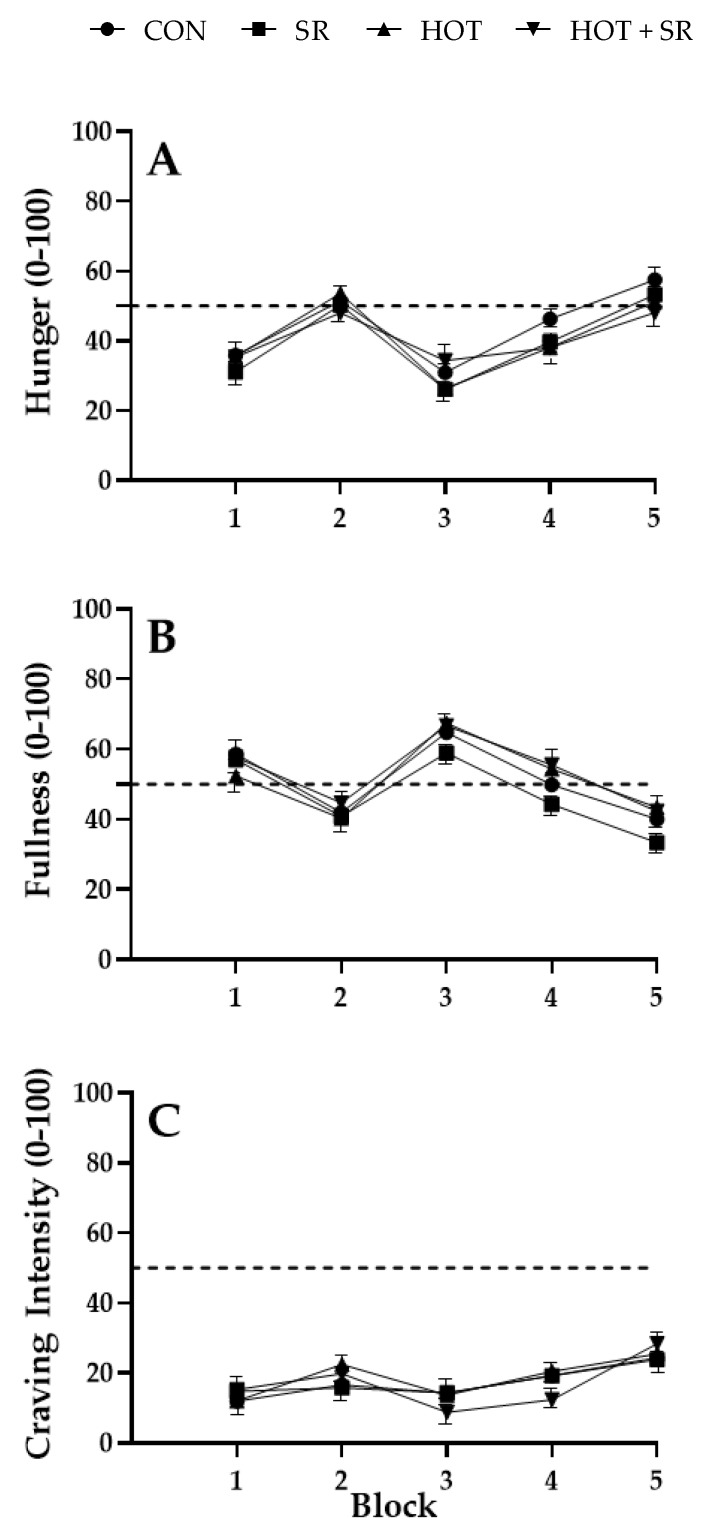
Ratings of hunger, fullness, and craving intensity across food opportunity blocks (1–5; *n =* 66). (**A**): hunger; (**B**): fullness; (**C**): craving intensity. CON = control condition. SR = sleep restricted condition. HOT = hot condition. HOT + SR = hot + sleep restricted condition. Block 1: 0800–1000; block 2: 1000–1200; block 3: 1230–1430; block 4: 1430–1630: block 5: 1630–1830. Lunch was provided at 1200 (after block 2) and dinner was provided at 1900 (after block 5). The dotted line represents the midline of the scale. Error bars indicate standard error.

A significant condition × block interaction was found for total energy intake (kJ; *p* = 0.002; Table 3 and Figure 2). In block 1 participants in the SR condition consumed less than participants in the CON condition. In block 3 participants in the SR condition consumed less than participants in the CON, HOT, and HOT + SR conditions. In block 4 participants in the HOT condition consumed more than participants in the SR and HOT + SR conditions. There was also a significant condition × block interaction for carbohydrate (g; *p* = 0.003; Table 3 and Figure 2). In block 1 participants in the SR condition consumed less than participants in the CON condition. In block 3 participants in the SR condition consumed less than participants in the CON, HOT, and HOT + SR conditions. In block 4 participants in the HOT condition consumed more than participants in the CON, SR, and HOT + SR conditions. There were no significant condition × block interactions for total fat (g) or protein (g; Table 3 and Figure 3). There were also no significant condition × day or condition × block interactions for total energy (kJ), carbohydrate (g), total fat (g), or protein (g; Table 3).

There was a significant main effect of the condition for total intake per food opportunity (Table 3) for total energy (kJ; *p* < 0.001), carbohydrate (g; *p* < 0.001), and total fat (g; *p* = 0.019). There was also a significant main effect of food opportunity block (Table 3) for total energy (kJ; *p* < 0.001), carbohydrate (g; *p* < 0.001), total fat (g; *p* < 0.001), and protein (g; *p* = 0.001). There was not a significant main effect of day (Table 3).

### 3.3. Ratings of Hunger, Fullness, and Craving Intensity

As seen in Table 3, there were no significant condition × day, condition × block (Figure 3), or day × block interactions for hunger, fullness, or craving intensity. There was a significant main effect of day for craving intensity (*p* = 0.015), but there was no significant main effect of block for hunger or fullness (Table 3). Significant main effects for food opportunity block were seen for hunger (*p* < 0.001), fullness (*p* < 0.001), and craving intensity (*p* < 0.001; Table 3). There were no significant main effects of condition for hunger, fullness, or craving intensity (Table 3). 

### 3.4. Ratings of Craving and Craving Type

Significant interactions between condition × block were not found for craving (Wald’s χ^2^ = 16.792, df = 12, *p* = 0.16), sweet cravings (Wald’s χ^2^ = 13.95, df = 12, *p* = 0.30), savoury cravings (Wald’s χ^2^ = 21.89, df = 12, *p* = 0.05), protein cravings (Wald’s χ^2^ = 14.20, df = 12, *p* = 0.29), or carbohydrate cravings (Wald’s χ^2^ =14.67, df = 12, *p* = 0.20). Likewise, no significant interactions were found between condition × day for craving (Wald’s χ^2^ = 1.96, df = 3, *p* = 0.58), sweet cravings (Wald’s χ^2^ = 1.67, df = 3, *p* = 0.65), savoury cravings (Wald’s χ^2^ = 3.55, df = 3, *p* = 0.31), protein cravings (Wald’s χ^2^ = 3.83, df = 3, *p* = 0.28), or carbohydrate cravings (Wald’s χ^2^ = 0.18, df = 3, *p* = 0.98). There was a significant main effect of block for sweet craving (Wald’s χ^2^ = 31.03 df = 4, *p* < 0.001), savoury cravings (Wald’s χ^2^ = 12.27, df = 4, *p* = 0.02), protein cravings (Wald’s χ^2^ = 31.36, df = 4, *p* < 0 0.01), and carbohydrate cravings (Wald’s χ^2^ = 12.25, df = 4, *p* = 0.02), with greater likelihood of reporting cravings in block 5. There was a significant main effect of the condition on sweet cravings (Wald’s χ^2^ = 8.92, df = 3, *p* = 0.03), with a greater likelihood of reporting sweet cravings in the control condition. No other main effect was significant.

## 4. Discussion

This is the first study to investigate the impact of sleep restricted to 4-h, and hot conditions, on food and macronutrient intake during snack opportunities, and the subjective ratings of hunger, fullness, and cravings of firefighters during multi-day simulated wildfire suppression. The findings suggest the timing of food intake differs under these conditions, rather than the total amount of food consumed. Specifically, firefighters consume more kilojoules and more carbohydrates between 1230–1430 when working in hot conditions after 4-h of sleep restriction, and only hot conditions; compared to working in only sleep restricted conditions and in control conditions. Further, sleep restriction and hot conditions did not alter subjective ratings of hunger, fullness, or craving intensity across the day.

There was no difference in total energy intake or total macronutrient intake from snacking between those that were sleep restricted, working in hot conditions, or sleep restricted and working in hot conditions in the current study. However, the timing of food intake differed across the day. These findings are consistent with multiple studies that report that the timing of food intake differed, but not the amount of food consumed, across multiple shift-types [25,58,59]. Of note, the current study is the first to suggest that this pattern of altered meal timing can be found within the same shift-type when worked under different environmental conditions. 

The most kilojoules were consumed during the food opportunity between 1230–1430 by those in the hot condition, the hot and sleep restricted condition, and the control condition, while those in the sleep restricted condition consumed the least number of kilojoules. This suggests that sleep restriction alone may not be enough to influence the food intake of firefighters during wildfire suppression, but hot conditions and the additive effect of hot conditions and sleep restriction has an influence on the amount of food eaten. An explanation for this finding may be energy expenditure. Although energy expenditure increases with sleep restriction leading to greater intake [45], physical activity can act as an appetite suppressant [60,61]. Our findings suggest that, for wildland firefighters, physical work may lead to decreased energy intake in sleep restricted conditions, compared to working in sleep restricted conditions with the addition of heat. The additive effect of heat on sleep restriction appears to increase energy intake, perhaps due to increased energy expenditure in hot conditions [42]. Further, that time of day (1230–1430) corresponds to the secondary window of circadian low when pressure for sleep is increased, and this biological sleep pressure would have been further exacerbated by sleep restriction [62,63]. Literature has shown an increase in snacking behaviour with increased sleepiness from sleep restriction [32,34,35] and with increased energy expenditure from sleep restriction [45,46]. Therefore, we may have expected that those in the sleep restricted conditions were more likely to consume the most kilojoules during this snacking period. Previously published results from this study have shown no difference in sleep quality between conditions, and confirmed that total sleep time was less in the sleep restricted condition [49]. Perhaps with a greater number of consecutive days of sleep restriction, or sleep deprivation (e.g. over 24-h awake), which are not uncommon for firefighters [17], we may see this pattern of greater food intake in the sleep restricted condition, although this remains speculative. 

Previous laboratory-based studies have shown increased snack consumption, including greater consumption of carbohydrates, when sleep-restricted [35,64]. Methodological differences may explain why this relationship was not observed in the current study. For example, previous laboratory studies that have explored food consumption in sleep restricted individuals often use shiftwork naïve participants [34,35] who may not be as familiar with sleep restriction as the experienced firefighters in the current study. For instance, those more familiar with sleep restriction may experience less of an acute effect of sleep restriction on food choice. This has implications for shiftworkers, as our knowledge about increased food consumption with sleep restriction from laboratory studies may only apply to those with limited shiftwork experience. Further, studies investigating food consumption after sleep restriction have not included physically demanding tasks and/or physical activity. In nonsleep restricted conditions, acute exercise does not influence immediate energy intake [37], but increased energy intake has been found 120 mins post-exercise, corresponding with increased ghrelin concentrations [65]. Current findings suggest this effect may be blunted with sleep restriction. This is important for understanding any changes in food intake in future research, as differences in food intake on-shift are not only impacted by prior sleep, but physical activity on-shift. 

Carbohydrate was the only macronutrient to differ based on condition, with higher carbohydrate consumption in the control condition, hot condition, and hot and sleep restricted condition compared to the sleep restricted condition between 1230–1430. Indeed, carbohydrate intake was lower during several blocks in the sleep restricted condition compared to other conditions. As such, the additive effect of the hot conditions and sleep restriction appears to influence carbohydrate intake. This may be due to increased stress levels and decreased alertness in response to these conditions, as in previous shiftwork literature, workers have reported increased carbohydrate consumption to increase alertness and feelings of comfort [25,66]. Further, previous findings have demonstrated increased carbohydrate utilisation during physical activity, particularly in hot conditions, and this has been linked to reduced fatigue [67]. In the current study, when participants were required to be physically active in hot conditions, while also sleep restricted, there may have been a greater drive for carbohydrates, compared to when they were physically active in only hot or sleep restricted conditions, or when they were in a control condition with no sleep restriction and a comfortable temperature. It should be noted that overall the ration pack items were lower in protein and fat than carbohydrates, which may have influenced the detection of between-group differences in protein and fat consumption across the study. 

Ratings of hunger and fullness showed the expected pattern based on the standard timing of food intake in Western cultures with increased hunger in the morning before lunch, decreased hunger after lunch, and increased hunger in the afternoon and evening [68]. This pattern also aligns with the circadian patterns of leptin and ghrelin, with leptin decreasing in the afternoon and evening, and ghrelin increasing before lunch and dinner times [30,31,69]. Interestingly, there were no differences in ratings of hunger and fullness between conditions, with participants in all conditions reporting similar levels across the day. Previous research has shown that sleep restricted individuals may report greater hunger during the day due to changes in levels of leptin and ghrelin [31,70]. However, the present results show no difference in the pattern of hunger in those who are sleep restricted. Participants in the current study in the sleep restricted and the hot and sleep restricted conditions had a 4-h sleep opportunity, and this is consistent with the sleep timing reported by firefighters [17]. We would expect to see sleep-related changes based on this level of sleep restriction. It is possible that leptin and ghrelin levels were altered by this sleep restriction but that this did not alter subjective levels of hunger, however this was not measured in the current study.

Of note, the increase in energy intake and carbohydrate intake from 1230–1430 did not correspond to an increase in ratings of hunger, and there were no differences in hydration between the conditions [49]. This suggests that this increase in food intake was not motivated by hunger, but perhaps by alertness, comfort, habit, or boredom. All factors that have previously been reported as influencing food intake on-shift in industries such as healthcare [71,72], factory work [73], transport [74], and aviation [75]. In addition, in one study of the eating behaviours of firefighters, a social eating culture was reported, with ‘communal cook-ups’ on-shift [47]. In the real-world, this social motivation for eating may be a greater influence on eating patterns than alertness and hunger. Taken together, these findings suggest that eating for reasons other than hunger is common amongst many shiftworking industries with unique shift demands and conditions, including firefighters. 

Overall there was no effect of sleep restriction or ambient temperature on cravings for food. While cravings increased towards the end of the day, following the pattern of hunger increasing and fullness decreasing at this time, this pattern occurred for all conditions and the intensity of these cravings were small. While the ration packs contained snacks that are commonly provided on-site for firefighters, perhaps these foods were not what the firefighters craved. In addition, this provides support for the argument that firefighters may be consuming food on-shift for reasons other than a craving to eat, for example for comfort or out of habit, as they were consuming food at these times, but were not experiencing strong cravings. 

This study is the first to demonstrate a difference in food intake for firefighters working under different conditions of sleep and heat, however there are some limitations. While firefighters were completing fireground tasks during the study, which were representative of actual fire suppression work, and were wearing their personal protective clothing, aspects of the laboratory environment may have limited external validity. For example, aspects of an outdoor environment such as wind, daylight, smoke, and sound, are likely to increase stress levels and potentially effect food intake in the real-world. Further, the participants were familiar with completing the fireground tasks under sleep restricted and hot conditions, and therefore may be more resilient to the effects of these conditions compared to workers in other industries. However, this can also be viewed as a strength of the study, as firefighters are under-represented in the shiftwork literature, and as this group face unique shift constraints, such as extreme temperatures, understanding the impact on food intake is important. Further investigation of the impacts of these conditions in a larger sample, and between males and females may also highlight individual differences in eating patterns. There are also some limitations to the nutritional data. The snacks in the ration packs were not replaced in each eating block but were replaced at the end of each day, and it is possible that there was a ceiling effect from certain snacks and if more of certain snacks were available then more may have been consumed. While the content of the breakfast, lunch, and dinner meals were representative, it is still possible that macro and micronutrient intake could vary. It should also be noted that sample size calculations for the larger study were based on the primary outcome measures which are published elsewhere [13,14,16,48,52]. 

To build on these findings there are several recommendations for future research. Measuring metabolic factors such as blood glucose, cholesterol, and serum hormone levels would be beneficial in understanding the physiological response to sleep restriction and hot conditions. Similarly, rigorous measures of energy expenditure under the different conditions (heat and sleep restriction) should be considered in future research, as this may impact snacking behaviour and cravings. Further, food intake motivation questionnaires have been used to understand why workers are choosing to eat at certain times [25]. Future research could utilise this questionnaire with firefighters to understand the motivations for eating during the day, as this is important for understanding what is driving eating behavior on-shift. The health effects of different snack choices, for example healthier snacks vs. high fat snacks, could also be investigated in future research in order to reduce the long-term health conditions reported by wildland firefighters [21,22]. Given firefighters are likely to be working into the night [17], replicating the current study during the night is an important next step. Completing the fire-suppression tasks at night, when the drive for sleepiness is greater [62] may lead to altered food timing and altered patterns of hunger compared to during the day, and provide important insights into the potential long-term health risks of eating at night. Understanding the sleepiness of participants pre- and post-bed would also be important for understanding how snack timing influences their mood. Future research could also measure drinking preference, for example are wildland firefighters likely to preference sweet beverages in different conditions. 

Firefighters are required to work long shifts in hot temperatures, often with restricted sleep, and understanding how this may impact food intake is important for ensuring that food can be supplied that is suitable to the conditions. Findings from this study suggest that it is not the amount of food that is altered in different conditions, but the timing of food intake. While future research is important to build on these results, this study has demonstrated the importance of understanding the food intake patterns of firefighters during wildfire suppression, and the need for more understanding of what motivates workers to eat on-shift, particularly with unique shift conditions. 

## Figures and Tables

**Table 1:** Participant demographics by study condition.

	Condition			
	CON (*n* = 18)	SR (*n* = 16)	HO T(*n* = 18)	HOT + SR (*n* = 14)
Age (y)	39 ± 16	39 ± 15	36 ± 13	41 ± 17
Male:Female	15:3	15:2	14:4	12:1
Body mass (kg)	85 ± 18	94 ± 20	88 ± 18	84 ± 14
Height (cm)	178 ± 8	178 ± 7	178 ± 9	176 ± 4
Body mass index (kg·m^2^)	27 ± 5	30 ± 6	28 ± 4	27 ± 4

Data are presented as mean ± SD. CON = Control; SR = Sleep restricted; HOT = Hot; HOT + SR = Hot and sleep restricted.

**Table 2:** Contents of firefighters’ ration pack during simulated wildfire suppression.

Snack (Quantity)	Total Energy (kJ) Per Serving	Total Protein (g) Per Serving	Total Fat (g) Per Serving	Total Carbohydrate (g) Per Serving
Granny smith apple (1)	309.0	0.5	0	16.1
Packet of Sunbeam sultanas (1)	532.0	1.0	0.4	27
Jatz crackers (3)	216.0	0.7	1.9	7.1
Packet of barbeque Shapes (1)	530.0	1.9	5.6	15.8
Packet of vegemite (1)	34.0	1.4	0.1	0.6
Arnott’s scotch finger biscuit (1)	380.0	1.1	3.8	11.7
Arnott’s nice biscuit (1)	216.0	0.7	1.7	8.8
Natural Confectionary Company snakes (5)	351.0	2.1	0.6	47.2
Uncle Toby’s raspberry yogurt top muesli bar (1)	517.0	2	5	19.5

Set meals were provided at 0630 (breakfast), 1200 (lunch), and 1900 (dinner), with the same quantities of food items available to all participants. For main meals, participants ate *ad libitum* from the available foods. Food items in the meals (breakfast, lunch and dinner) were based on the food items that would be available to firefighters on the fireground [47]. These meals were representative in terms of food type, quantity, and variety, and included a hot breakfast of toast and eggs, sandwiches for lunch, and a hot dinner that included pasta, meat, and vegetables [52].

**Table 3:** Results of mixed effect ANOVAs, showing the main effects of condition, day, and food opportunity block, and the interaction between condition × day, condition × block, and condition × day for total intake per day from snack opportunities and total intake per food opportunity.

	Condition		Day		Block		Condition × Day		Condition × Block		Day × Block	
	*F_(df)_*	*p*	*F_(df)_*	*p*	*F_(df)_*	*p*	*F_(df)_*	*p*	*F_(df)_*	*p*	*F_(df)_*	*p*
*Total Intake Per Day from Snack Opportunities* (*n* = 50)												
Total Energy (kJ)	1.16_(3, 50.86)_	0.333	0.14_(1, 41.23)_	0.710	-	-	2.21_(3, 43.65)_	0.100	-	-	-	-
Carbohydrate (g)	1.42_(3, 51.40)_	0.248	0.05_(1, 41.05)_	0.842	-	-	1.93_(3, 43.31)_	0.140	-	-	-	-
Total Fat (g)	0.98_(3, 50.96)_	0.409	0.02_(1, 46.04)_	0.897	-	-	0.66_(3, 49.91)_	0.583	-	-	-	-
Protein (g)	1.21_(3, 85.00)_	0.313	1.35_(1, 85.00)_	0.248	-	-	1.23_(3, 85.00)_	0.303	-	-	-	-
*Total Intake Per Snack Opportunity* (*n* = 50)												
Total Energy (kJ)	11.52_(3, 435.00)_	< 0.001 *	1.08_(1, 435.00)_	0.300	18.57_(4, 435.00)_	< 0.001 *	0.34_(3, 435.00)_	0.797	2.55_(12, 435.00)_	0.003 *	0.22_(4, 435.00)_	0.130
Carbohydrate (g)	11.89_(3, 435.00)_	< 0.001 *	1.23_(1, 435.00)_	0.268	18.273_(4, 435.00)_	< 0.001 *	0.26_(3, 435.00)_	0.854	2.40_(12, 435.00)_	0.01 *	0.32_(4, 435.00)_	0.866
Total Fat (g)	3.15_(3, 47.93)_	0.033 *	2.03_(1, 408.89)_	0.155	8.67_(4, 392.63)_	< 0.001 *	0.31_(3, 407.50)_	0.819	1.59_(12, 392.63)_	0.093	0.39_(4, 392.63)_	0.819
Protein (g)	1.77_(3, 47.81)_	0.165	1.46_(1, 406.50)_	0.228	4.29_(4, 391.98)_	0.002 *	0.51_(3, 405.17)_	0.67	1.42_(12, 4391.89)_	0.154	0.33_(4, 391.89)_	0.984
*Ratings of Hunger, Fullness, and Craving Intensity* (*n* = 66)												
Hunger	0.43_(3, 83.14)_	0.732	2.09_(1, 739.80)_	0.149	79.07_(4, 738.21)_	< 0.001 *	0.39_(3, 739.78)_	0.762	1.67_(12, 738.21)_	0.069	1.55_(4, 738.21)_	0.186
Fullness	0.79_(3, 82.83)_	0.502	0.11_(1, 739.85)_	0.745	71.30_(4, 737.92)_	< 0.001 *	0.55_(3, 739.83)_	0.651	1.64_(12, 737.92)_	0.076	2.15_(4. 737.92)_	0.073
Craving Intensity	0.02_(3, 83.09)_	0.996	6.00_(1, 731.28)_	0.015*	12.23_(4, 730.21)_	< 0.001 *	2.10_(3, 731.27)_	0.099	0.88_(12, 730.21)_	0.568	1.17_(4, 730.26)_	0.322

Note. * *p* < 0.05.

**Table 4:** Total intake per day from snacking opportunities (*n* = 50) for energy (kJ), carbohydrate, total fat, and protein.

	Total Energy (kJ)	Carbohydrate (g)	% of Total Energy	Total Fat (g)	% of Total Energy	Protein (g)	% of Total Energy
CON							
Day 2	1935.37 ± 208.17	82.12 ± 8.49	71.01	13.76 ± 3.37	11.90	9.08 ± 3.05	17.67
Day 3	1552.73 ± 208.15	68.43 ± 8.49	73.76	16.53 ± 3.37	17.81	12.98 ± 3.05	31.4
SR							
Day 2	1558.73 ± 208.15	56.10 ± 7.64	60.23	8.04 ± 3.03	8.63	4.71 ± 2.74	11.38
Day 3	1733.72 ± 200.01	70.12 ± 8.15	67.69	11.44 ± 3.24	11.04	6.44 ± 2.93	13.99
HOT							
Day 2	1333.46 ± 187.50	62.32 ± 9.66	78.22	10.86 ± 3.84	13.63	5.17 ± 3.47	14.60
Day 3	1733.72 ± 200.01	73.19 ± 9.65	70.65	12.58 ± 3.84	12.14	7.03 ± 3.47	15.27
HOT + SR							
Day 2	1469.06 ± 250.68	60.64 ± 10.22	69.08	12.95 ± 4.04	14.75	6.28 ± 3.66	16.10
Day 3	1039.09 ± 92.01	44.77 ± 10.73	72.11	6.43 ± 4.29	10.36	3.86 ± 3.88	13.99

Note. CON = control condition. SR = sleep restricted condition. HOT = hot condition. HOT + SR = hot + sleep restricted condition. All results presented are estimated marginal means ± standard error.
